# Body Composition of Elite Female Players in Five Different Sports Games

**DOI:** 10.1515/hukin-2015-0021

**Published:** 2015-04-07

**Authors:** Lucia Mala, Tomas Maly, František Zahalka, Vaclav Bunc, Ales Kaplan, Radim Jebavy, Martin Tuma

**Affiliations:** 1 Sport Research Center, Charles University in Prague, Prague, Czech Republic.; 2 Department of track and field, Charles University in Prague, Prague, Czech Republic.; 3 Department of sports games, Charles University in Prague, Prague, Czech Republic.

**Keywords:** testing, females, fat mass, fat-free mass, athletes, bioimpedance analysis

## Abstract

The goal of this study was to identify and compare body composition (BC) variables in elite female athletes (age ± years): volleyball (27.4 ± 4.1), softball (23.6 ± 4.9), basketball (25.9 ± 4.2), soccer (23.2 ± 4.2) and handball (24.0 ± 3.5) players. Fat-free mass (FFM), fat mass, percentage of fat mass (FMP), body cell mass (BCM), extracellular mass (ECM), their ratio, the percentage of BCM in FFM, the phase angle (α), and total body water, with a distinction between extracellular (ECW) and intracellular water, were measured using bioimpedance analysis. MANOVA showed significant differences in BC variables for athletes in different sports (F60.256 = 2.93, p < 0.01, η2 = 0.407). The results did not indicate any significant differences in FMP or α among the tested groups (p > 0.05). Significant changes in other BC variables were found in analyses when sport was used as an independent variable. Soccer players exhibited the most distinct BC, differing from players of other sports in 8 out of 10 variables. In contrast, the athletes with the most similar BC were volleyball and basketball players, who did not differ in any of the compared variables. Discriminant analysis revealed two significant functions (p < 0.01). The first discriminant function primarily represented differences based on the FFM proportion (volleyball, basketball vs. softball, soccer). The second discriminant function represented differences based on the ECW proportion (softball vs. soccer). Although all of the members of the studied groups competed at elite professional levels, significant differences in the selected BC variables were found. The results of the present study may serve as normative values for comparison or target values for training purposes.

## Introduction

Body composition (BC) is an important indicator of the physical fitness and health of athletes. Excess adipose tissue acts as dead weight in activities during which the body mass must be repeatedly lifted against gravity during locomotion and jumping (Reilly, 1996); this in turn decreases performance and increases the energy demands of the activity. In contrast, fat-free mass contributes to the production of power during high-intensity activities and provides greater absolute strength for resistance to high dynamic and static loads.

Many team sports (basketball, soccer and handball) are characterised by intermittent loading during the match–play. In volleyball, explosive strength of the lower limbs is important for jump height. At the elite level, anthropometric and BC variables are monitored to compare players of different positions (Carter et al., 2005; Malousaris et al., 2008; Sedano et al., 2009), successful and unsuccessful teams (Carter et al., 2005; Maly et al., 2011) and teams with lower and higher performance levels (Bayios et al., 2006; Malousaris et al., 2008; Sedano et al., 2009).

Although a number of published studies have investigated specific BC variables, there are fewer studies of female athletes than of male ones. A smaller number of studies have focused on sports involving game play (Carbuhn et al., 2010; Sedano et al., 2009); however, to the best of our knowledge, only a few studies have reported comparisons of BC among elite female athletes (Bayios et al., 2006; Fleck, 1983; Gholami and Rad, 2010). One study described the BC profiles of elite female players on one team (Maly et al., 2010), while others have compared BC profiles between elite handball (Hasan et al., 2007) and volleyball (Maly et al., 2011) teams; yet other studies have examined the relationships between BC variables and health or performance (Melrose et al., 2007; Sedano et al., 2009).

Many studies of BC in athletes focus on relative fatness because of the potentially negative influence of excessive fat on performance. From a methodological point of view, it is difficult to compare the BC variables of elite female players from different studies due to differences in the methods used to assess BC variables, performance levels, and in diagnostic time (different phases of annual training cycle). Because of these limitations, we compared BC in elite female athletes from five different team sport games under same conditions before the main event of the season. The presented profile and comparisons include a wider range of variables identifying BC than those in other available studies.

## Material and Methods

### Participants

The sample consisted of elite female players (n = 80) of five team sports games: volleyball (V), softball (S), basketball (B), soccer (SC) and handball (H). The performance level profiles of the tested teams are shown in Table 1 and anthropometric variables in Table 2.

### Body composition assessment

Body composition variables were monitored during a training session in preparation for the national team seasons (3–5 days before the event), with the exception of the volleyball players, who were examined during the group stage of the Champions league play (3 days before the match in the Champions League). Measurements were performed in the morning before breakfast (7:30 – 8:30 AM) by the same examiner in all monitored groups. BC was measured using the bioimpedance method under the standard conditions described in the BIA guidelines (Kyle et al., 2004).

In the 24 hours prior to the measurements, the participants did not consume any medications (including alcohol and caffeine) or pharmacological agents that could influence the results of the measurement. They were also told not to eat or drink before the measurement. Furthermore, 48 hours before the tests the players did not perform high intensity physical activity. All tests were carried out during the estrogenic phase of the menstrual cycle.

Body mass (BM) was measured by a digital scale (SECA 769, Hamburg, Germany) and body height (BH) by a digital stadiometer (SECA 242, Hamburg, Germany).

Whole-body bioimpedance was measured with the phase-sensitive whole-body tetrapolar bioimpedance measurement device (BIA 2000 M, Data Input GmbH, Germany). We registered the following BC variables: fat-free mass (FFM), percentage of fat mass (FMP), absolute fat mass (FMA); body cell mass (BCM), extracellular mass (ECM) and their mutual ratio (ECM/BCM); the percentage of BCM in FFM (BCMP), the phase angle (α), and total body water (TBW), with a distinction between extracellular (ECW) and intracellular (ICW) water. To detect FFM, we used the equation as described by Fornetti et al. (1999).

The participants received a verbal description of the study procedures before testing and completed a written informed consent form that was approved by the ethical committee of Physical Education and Sport, Charles University in Prague. Measurements were performed according to the ethical standards of the Helsinki Declaration and the ethical standards in sport and exercise science research described by Harriss and Atkinson (2011).

### Statistical analysis

Differences in the selected body composition parameters among the observed groups were assessed by multivariate analysis of variances (MANOVA). We used the Levene’s test of equality of error variances to verify the assumption that the error variance of the dependent variable was equal across categories. We used a multiple comparison of means (Bonferroni’s post–hoc test) to compare differences in particular parameters among individual groups.

The rejection of the null hypothesis was assessed at the level of *p* ≤ 0.05. Effect size was assessed using the “Eta square” coefficient (*η^2^*), which explains the proportion of variance of the monitored factor. Effect size was examined as follows: *η^2^* = 0.20 – small effect, *η^2^* = 0.50 – medium effect and *η^2^* = 0.80 – large effect (Cohen, 1992). Discriminant function analysis (stepwise criteria) was then applied to 11 variables of body composition.

## Results

MANOVA showed significant differences in body composition variables between athletes from different team sports (*F*_60,256_ = 2.93, *p* < 0.01, *η^2^* = 0.407). Comparison of basic parameters revealed significant differences in players age (*F*_4,75_ = 3.12, *p* < 0.02, *η^2^* = 0.143). Bonferroni’s post–hoc tests indicated a significant difference between female volleyball and soccer players (*p* < 0.05). We also found differences in players height (*F*_4,75_ = 24.29, *p* < 0.01, *η^2^* = 0.564; post–hoc test: V vs. S, SC, or H: *p* < 0.01; B vs. S, SC, or H: *p* < 0.01; SC vs. H: *p* < 0.01), players body mass (*F*_4,75_ = 9.59, *p* < 0.01, *η^2^* = 0.338; post–hoc test: SC vs. V, B, or H: *p* < 0.01; S vs. B: *p* < 0.01) and the BMI (*F*_4,75_ = 4.33, *p* = 0.003, η*^2^* = 0.188; post–hoc test: V vs. S or H *p* < 0.01) between female athletes in different team sports games.

The BC profiles and differences in the selected BC variables among the groups of elite female athletes are presented in Table 3.

**Figure 1:**
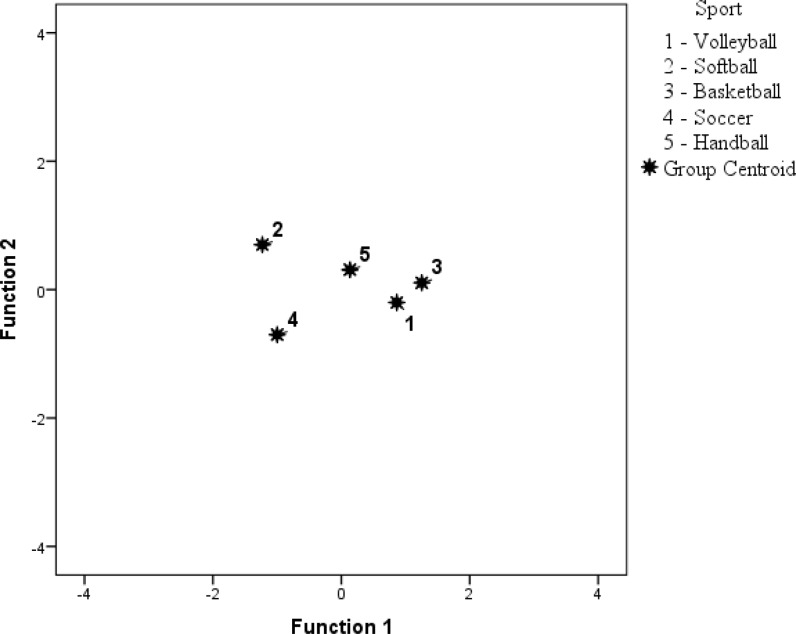
Canonical discriminant functions

Discriminant analysis revealed two significant functions (*p < 0.01*) (Table 4). The first discriminant function primarily represented differences based on the FFM proportion (volleyball, basketball vs. softball, soccer). The second discriminant function represented differences based on the ECW proportion (softball vs. soccer). All of the variance explained by the model is due to the first two discriminant functions. Based on values of Wilk’s lambda, the first discriminant function accounted for 80.5% (eigenvalue = 0.998) of the total variance, while the second discriminant function explained 19.5% (eigenvalue = 0.242) of the remaining variance. Figure 1 and Table 5 represent group centroid distances between groups for both discriminant functions.

## Discussion

The recorded mean values of BH, BM or the BMI (Table 2) are consistent with previously reported values for elite athletes. Lower BH in elite female soccer and softball players and the possibility of higher proportions of FFM in “taller players” could contribute to the significant differences between the compared parameters in tested groups, at least those expressed as absolute values [FFM, ECM, BCM, FMA and TBW, ICW and ECW (Table 3)]. Neither relative inactive mass proportion (FMP) nor α exhibited significant differences or size effects between the tested teams.

In case of FFM, we observed significant differences between the compared disciplines. Soccer players achieved significantly lower FFM values than those of volleyball, basketball and handball players (Table 3). A significant difference was also found between softball and basketball players. These differences could also be caused by the difference in BH between sports.

A comparison of absolute FFM values that also accounted for somatometric variables and the relative values of other parameters described below indicated that the softball players had the best active FFM (despite lower body mass, players had relatively high absolute FFM); surprisingly, the athletes with the poorest active FFM were the basketball players (Table 3). BCM, as a part of FFM, is defined as a predictor of muscle efficiency for sport performance (Andreoli et al., 2003). The level of BCM was significantly lower in female soccer players compared to the other monitored athletes. The differences in percentage between female soccer players and other female athletes were as follows: SC vs. V = 10.3%, SC vs. S = 11.5%, SC vs. B = 10.5% and SC vs. H = 10.5%. This difference may be attributed to the lower BH or BM in soccer players, which could affect the total FFM and its components compared to other teams.

The percentage of body cell mass in FFM (BCM_P_), an indicator of physical fitness and nutrition state of athletes, was highest in female softball players, while female basketball players had the lowest proportion (percent difference between S and B = 12.3%).

Analysis of individual players reveals values approaching the recommended 50% in the softball and soccer teams. BCM_P_ can be as high as 60% FFM in elite athletes (Dörhöfer and Pirlich, 2007), and in female volleyball players, values of approximately 50% are common (Mala et al., 2010). The smaller deficit in BCM as a proportion of FFM in individual female athletes may be caused by individual differences; it may also be the result of different adaptations to the different types of training for different sports (for instance, strength training frequency) or the difference in the physiological demands on the players from the sport itself (i.e., during match–play). These hypotheses should be verified by general and specific motor tests and analyses of training loads in particular disciplines should be considered.

Another variable with a high informative value is α, which directly links intracellular liquid to BCM (Malá et al., 2010). De Lorenzo et al. (1997) define BCM as the total mass of the “oxygen– exchanging, potassium–rich, glucose–oxidizing, work–performing” cells of the body. BCM is considered the actively metabolising portion of the FFM. A comparison of individual components of FFM showed that the average proportion of BCM was higher than that of ECM in all tested players.

The recorded phase angle values indicate high cell density and a high proportion of intracellular volume in all monitored players. The phase angle is an indicator of cellular health and integrity. A low α indicates that cells are unable to store energy, reflecting a breakdown in the selective permeability of cellular membranes. A high α is consistent with large quantities of intact cell membranes and BCM (Ceritsuat et al., 2010). No significant differences between teams were observed, possibly due to the heterogeneity of the tested group, as reflected by the large standard deviation values (Table 3).

Bayios et al. (2006) emphasise the variability of BC among athletes in different sports. The authors found a higher FMp in female handball players than basketball and volleyball players. In contrast, Gholami and Rad (2010) did not observe significant differences in absolute FM_A_ and FM_P_ of female basketball and handball players from the Iranian national teams, nor did they find differences in the players BMI or BM. Carbuhn et al. (2010) reported insignificant differences in FM_P_ between female softball, basketball and volleyball players during the off-season and post-season periods. However, after the pre-season period, they observed significantly higher FM_P_ in volleyball players than in softball and basketball players. The authors also reported insignificant changes in FMP during the season in volleyball. In contrast, during the pre-season period, softball and basketball players showed significant decreases in FMP. The absence of significant differences in FM_P_ between the teams demonstrates that all athletes make an effort to eliminate inactive mass, regardless of sport. Future studies should focus on FM_P_, which is currently considered not only a crucial variable for the assessment of an individual’s fitness level but also eventual health risks (Fruth et al., 2008).

The FFM variable stands for the first discriminant function which primarily represented differences between groups (volleyball, basketball vs. softball, soccer). The reason would be greater body height and mass of female volleyball and basketball players in comparison to female softball and soccer players. FFM is highly correlated with overall body size (Malina, 2007).

The second discriminant function represented differences based on the ECW proportion (softball vs. soccer). The basis for ECW discrimination is in the following matters. ECW difference between female softball and soccer players is 16%. Female softball players have the highest BMI index (23.5 ± 2.8 kg/m^2^) among all the tested groups while female soccer players the second from bottom (21.9 ± 1.4 kg/m^2^). Despite similar body height (difference between groups was 1.5%) female softball players exhibited higher values of body mass (9.7%) and FM_A_ (23%) and only 7.4% in FFM (in favour of softball players). Ritz et al. (2008) reported that the greater the fat mass the greater the extra-cellular compartment. Female softball players show greater „body shape“ and therefore, also a higher proportion of ECW in comparison to female soccer players. ECW is composed of water in support and transport tissues: plasma, dense connective tissue (tendon, cartilage, bone), interstitial lymph, and transcellular fluids (cerebrospinal fluid, joint fluids). ICW corresponds closely to skeletal muscle mass, the work producing tissue of the body (Malina, 2007).

Despite explained overall variability of the model using two discriminant functions it is difficult to correctly classify the group of female handball and softball players based on the monitored variables (Table 6). When looking at the monitored sport disciplines, we have to consider different motor and metabolic demands of the studied disciplines. For instance, soccer requires a significant level of aerobic endurance as female athletes cover 9292 ± 175 m (Hewitt et al., 2014) during a game. Softball and volleyball are anaerobic in nature and speed and power are the dominant motor abilities in contact sports like handball and basketball where size and strength may help in fighting for the position on the court and where athletes may also benefit from higher BM and BH.

For further research it would be necessary to find another predictor in order to separate these players. A suitable solution would be to include other predictors, namely anthropometric and motor variables (Leone et al., 2002), a competition level (Coelho e Silva et al., 2010), and team’s success (Dimitros et al., 2013).

## Conclusion

Despite the elite, professional status of all of the examined athletes, significant differences in the selected BC variables were observed between groups. No significant changes in FM_P_ or the phase angle (α, an indicator of cellular health and integrity) were detected between the monitored groups. The type of sport, as an independent variable, had a significant effect on other BC variables.

The athletes with the most distinct BC were soccer players, who differed from players of at least one other sport in 8 out of 10 compared variables. In contrast, volleyball and basketball players did not differ significantly from each other in any of the compared BC variables. Discriminant analysis revealed two significant functions (FFM, ECW); however, for better distinction between the monitored groups it is necessary to search for other predictors.

The descriptive results presented in this study can serve as normative values for comparison or as target values for a training regimen design. However, researchers and coaches must always consider a performance level, methods used and the time of measurement within the periodisation of sports training when interpreting these and other results.

## Figures and Tables

**Table 1:** Performance levels of the tested athletes

Sport	Club / National team	Performance level
Volleyball	Club	Winner of the national league and league cup, participant in the highest level of the European Cup Championship CEV^1^, winner of Middle European league MEVZA^2^
Softball	National team	Participant in World Championship play
Basketball	National team	Second place in World Championship competition (silver medal)
Soccer	National team	Participant in European Championship play
Handball	National team	Qualified for European Championship play

1 – Confédération Européenne de Volleyball

2 – Middle European Volleyball Zonal Association

**Table 2:** Basic anthropometric variables of the tested players (mean ± SD)

Sport	n	Age (years)	Body height (cm)	Body mass (kg)	BMI (kg·m^−2^)
Volleyball	18	27.4 ± 4.1	184.3 ± 4.2	72.1 ± 6.5	21.2 ± 1.4
Softball	14	23.6 ± 4.9	169.9 ± 7.1	67.9 ± 9.9	23.5 ± 2.8
Basketball	14	25.9 ± 4.2	185.8 ± 9.0	76.6 ± 7.8	22.2 ± 1.5
Soccer	18	23.2 ± 4.2	167.3 ± 6.8	61.3 ± 5.5	21.9 ± 1.4
Handball	16	24.0 ± 3.5	176.0 ± 6.5	72.5 ± 8.3	23.4 ± 2.3
Total	80	24.9 ± 4.4	176.6 ± 10.0	69.8 ± 9.1	22.4 ± 2.1

BMI – body mass index

**Table 3:** Body composition profiles and differences in elite female athletes from different team sports

Variables		**Volleyball**	**Softball**	**Basketball**	**Soccer**	**Handball**	**Univariate ANOVA**		**Bonferroni’s post**–**hoc test**
		**n = 18**	**n = 14**	**n = 14**	**n = 18**	**n = 16**	***F***	η*^2^*	
TBW (l)	a	39.89 ± 2.96	39.11 ± 4.20	41.20 ± 3.03	35.47 ± 2.51	39.88 ± 3.44	7.64^**^	0.29	SC vs. V, B, H^**^
	b	34.70 – 46.20	33.20 – 45.20	35.00 – 46.30	30.20 – 40.20	34.00 – 45.30			SC vs. S^*^
ICW (l)	a	23.44 ± 0.92	22.99 ± 1.31	23.66 ± 0.97	21.93 ± 0.86	23.01 ± 1.00	7.15^**^	0.28	SC vs. V, B^**^
	b	21.50 – 24.40	21.00 – 24.70	21.50 – 25.50	20.10 – 23.30	21.20 – 24.70			SC vs. S, H^*^
ECW (l)	a	16.54 ± 2.07	16.10 ± 2.93	17.59 ± 2.25	13.52 ± 1.69	16.79 ± 2.49	7.7^**^	0.29	SC vs. V B, H^**^
	b	13.20 – 20.80	12.10 – 20.50	13.50 – 21.80	10.10 – 16.90	12.80 – 20.60			SC vs. S^*^
FFM (kg)	a	57.81 ± 4.53	53.35 ± 5.74	60.30 ± 5.42	49.40 ± 4.31	56.95 ± 5.34	11.91^**^	0.39	SC vs. V, B, H^**^
	b	48.57 – 65.97	45.24 – 62.33	50.12 – 69.50	41.99 – 55.92	46.84 – 64.95			S vs. B^**^
ECM (kg)	a	23.96 ± 1.87	22.47 ± 2.41	24.54 ± 2.03	21.07 ± 1.68	23.89 ± 2.52	7.44^**^	0.28	SC vs. V, B, H^**^
	b	21.30 – 27.00	19.60 – 26.80	21.80 – 27.20	18.10 – 24.60	20.00 – 27.70			
BCM (kg)	a	30.53 ± 2.77	30.94 ± 3.69	30.61 ± 2.71	27.39 ± 2.16	30.59 ± 3.33	4.36^**^	0.19	SC vs. V, S, B, H^*^
	b	25.00 – 36.90	25.40 – 37.00	25.20 – 34.90	23.20 – 30.90	24.30 – 35.40			
ECM/BCM	a	0.79 ± 0.07	0.73 ± 0.05	0.80 ± 0.06	0.77 ± 0.06	0.79 ± 0.10	7.44^**^	0.28	S vs. B^*^
	b	0.70 – 0.92	0.62 – 0.80	0.70 – 0.90	0.68 – 0.90	0.63 – 0.97			
BCM_P_ (%)	a	42.39 ± 2.19	45.82 ± 3.66	40.20 ± 4.09	44.82 ± 3.28	42.32 ± 3.22	6.79^**^	0.27	B vs. S, SC^**^
	b	38.35 – 46.14	38.37 – 51.90	28.33 – 45.22	40.03 – 53.26	37.41 – 47.21			S vs. V, H^*^
α (°)	a	6.91 ± 0.48	7.36 ± 0.46	6.81 ± 0,43	7.02 ± 0.45	6.96 ± 0.76	2.24	0.11	-
	b	6.00 – 7.60	6.80 – 8.40	6.10 – 7.60	6.10 – 7.80	5.80 – 8.30			
FM_P_ (%)	a	19.77 ± 1.77	21.35 ± 3.67	21.22 ± 1.66	19.53 ± 2.59	21.43 ± 2.48	2.27	0.11	-
	b	17.13 – 22.56	17.51 – 30.80	18.72 – 25.60	14.66 – 24.30	16.44 – 25.86			
FM_A_ (kg)	a	14.33 ± 2.32	14.76 ± 4.53	16.34 ± 2.72	12.01 ± 2.10	15.59 ± 3.27	4.89^**^	0.21	SC vs. B, H^**^
	b	10.43 – 18.28	9.98 – 26.50	12.36 – 22.52	7.65 – 16.21	9.88 – 22.65			

TBW – total body water, ICW – intracellular water, ECW – extracellular water, FFM – fat free mass, ECM – extracellular mass, BCM – body cell mass, ECM/BCM – extracellular-intracellular mass ratio, BCM_P_ –percent BCM in FFM, α – phase angle, FM_P_ – percent fat mass, FM_A_ – absolute fat mass,

a – mean ± standard deviation,

b – range,

* –p < 0.05,

^**^ – p < 0.01

**Table 4:** Results of stepwise discriminant analyses

**Step**	**Entered**	**Wilks’ Lambda**	**df1**	**df2**	**df3**	**Exact F**	**df1**	**df2**	**Sig.**
1	FFM (kg)	0.61	1	4	75	11.91	4	75	0.00
2	ECW (l)	0.40	2	4	75	10.64	8	148	0.00

FFM – fat free mass, ECW – extracellular water

**Table 5:** Group centroids for two discriminant functions

SPORT	Group centroids	

	DF1	DF2
Volleyball	0.86	−0.20
Softball	−1.23	0.70
Basketball	1.25	0.11
Soccer	−1.00	−0.70
Handball	0.13	0.31

DF – discriminant function

**Table 6:** Classification for all discriminant functions after validation

Sport	Predicted group membership, n (%)					

	*n*	Volleyball	Softball	Basketball	Soccer	Handball
Volleyball	18	11 (61.1)	0	3 (16.7)	2 (11.1)	2 (11.1)
Softball	14	0	7 (50.0)	0	6 (42.9)	1 (7.1)
Basketball	14	3 (21.4)	0	7 (50.0)	1 (7.1)	3 (21.4)
Soccer	18	2 (11.1)	1 (5.6)	0	12 (66.7)	3 (16.7)
Handball	16	3 (18.8)	2 (12.5)	2 (12.5)	4 (25.0)	5 (31.3)

Percent of correctly classified cases: 52.5 %
